# Development and Validation of the ‘Working With Chronic Pain: Assessment of Impacts’ (WORC‐PAIN) Questionnaire for People in Paid Work With Chronic Pain

**DOI:** 10.1002/ejp.70337

**Published:** 2026-07-29

**Authors:** LaKrista Morton, Martin J. Stevens, R. Stuart Anderson, Nicola Goodson, Ira Madan, M. M. Suzanne Verstappen, Elaine Wainwright, Karen Walker‐Bone, Gary J. Macfarlane, Rosemary Hollick

**Affiliations:** ^1^ Arthritis UK/Medical Research Council Centre for Musculoskeletal Health and Work, University of Aberdeen Aberdeen UK; ^2^ Arthritis UK/Medical Research Council Centre for Musculoskeletal Health and Work, Liverpool John Moores University Liverpool UK; ^3^ Arthritis UK/Medical Research Council Centre for Musculoskeletal Health and Work, Guy's & St Thomas' NHS Foundation Trust London UK; ^4^ Arthritis UK/Medical Research Council Centre for Musculoskeletal Health and Work, University of Manchester Manchester UK; ^5^ Monash Centre for Occupational and Environmental Health, Monash University Melbourne Victoria Australia

## Abstract

**Background:**

Chronic pain (CP) impacts people's work in a variety of ways but no instrument captures the totality of these impacts. This study aimed to develop and validate a new instrument, ‘Working with Chronic Pain: Assessment of Impacts’ (WORC‐PAIN).

**Methods:**

We employed a sequential mixed‐methods design: 1. Focus groups with people with CP and other stakeholders to identify key domains of work affected by CP; 2. A multi‐stage Delphi process to refine items based on identified domains; 3. Testing WORC‐PAIN within a cohort of people working with CP and analysing responses using factor analysis to identify subscales which were tested for internal consistency, concurrent validity, and test–retest reliability. Concurrently, item comprehension was evaluated via a Think Aloud process.

**Results:**

50 focus group participants defined domains for inclusion. Items were drafted based on domains, and input from 12 Delphi Stage 1 and 55 Delphi Stage 2 participants further refined these. 919 people with CP completed the WORC‐PAIN questionnaire and nine participated in the Think Aloud process. Three key domains were identified from the factor analysis: wider impacts of working with CP, day‐to‐day impacts, and individual and job‐based facilitators to support working with CP. Subscales were generated for each domain and were shown to have good internal consistency (*α* = 0.60–0.89), convergent validity, and test–retest reliability (intraclass correlation coefficients = 0.82–0.92).

**Conclusions:**

WORC‐PAIN is a new instrument, developed with people living with CP, which can quantify effects of CP on work and which will facilitate interventions to better support people living with CP to work.

**Significance:**

This article presents on the development and validation of a new instrument to measure impacts of chronic pain on work. Over 100 people with experience of working with chronic pain and other key stakeholders were involved in its development, and the new ‘Working with Chronic Pain: Assessment of Impacts’ (WORC‐PAIN) instrument can validly and reliably measure impacts that are important to people working with chronic pain at a population‐level.

## 1 Introduction

Chronic pain is common and affects physical and mental health (Breivik et al. 2006; Kawai et al. 2017). It not only limits engagement in family and social life, but also participation in paid work (Briggs et al. 2016; Dueñas et al. 2016; Hadi et al. 2019; Hengstebeck et al. 2017; Solomon et al. 2007). Work that is ‘healthy, safe, and [that] offer[s] the individual some influence over how work is done’ is important for physical and mental health, and overall well‐being (Black 2008). So‐called ‘good work’ contributes to an individual's sense of identity and wider contribution to society, and enables financial independence (Black 2008; Waddell and Burton 2006).

Given the importance of ‘good work’, which includes principles of ‘decent work’ as defined by the International Labour Organization and European Union (e.g., dignity, safety, security) (European Commission 2022; International Labour Organization 2019), and the impacts that chronic pain can have on work, it is important for researchers to accurately measure these impacts from the perspective of both persons living with chronic pain and employers. This would enable the identification of issues which are important to people with pain, to ultimately be able to identify targeted solutions, and assess whether solutions have been successful. Sickness absence was originally used as the measure of the impact of chronic pain on work, and is still often used as its primary indicator (Stagg et al. 2022). More recently, measures of presenteeism have been used (Hemp 2004; Stagg et al. 2022). However, these measures are limited to assessing aspects related to the quantity of work (Ravinskaya et al. 2022) and fail to capture wider impacts that are important to people working with chronic pain (Stagg et al. 2022).

The present study reports on the Quantifying the Impact of Chronic pain on paid worK (QUICK) study, which aimed to develop a multidimensional instrument for quantifying impacts of chronic pain on paid work at a population level and in intervention settings. A systematic review was conducted as the first part of the QUICK study to identify how impacts of chronic pain on engagement in work have been measured quantitatively, and to contrast these with the qualitative impacts that people working with chronic pain have described (Stagg et al. 2022). While the qualitative literature touched on all the identified themes, those addressed by existing quantitative instruments were limited (Stagg et al. 2022).

The aim of this exploratory sequential mixed‐methods study (Creswell and Clark 2017) was to develop and refine a methodologically robust instrument that captures impacts of chronic pain that are important to people working in paid work with pain and other stakeholders. This study aimed to:

- Identify key domains to include in a new instrument to assess impacts of chronic pain on work;
- Develop an instrument with face and content validity for people working with chronic pain and other stakeholders;
- Validate the new instrument and report on its scoring, internal consistency, convergent validity, and test–retest reliability.

## 2 Methods

**FIGURE 1:**
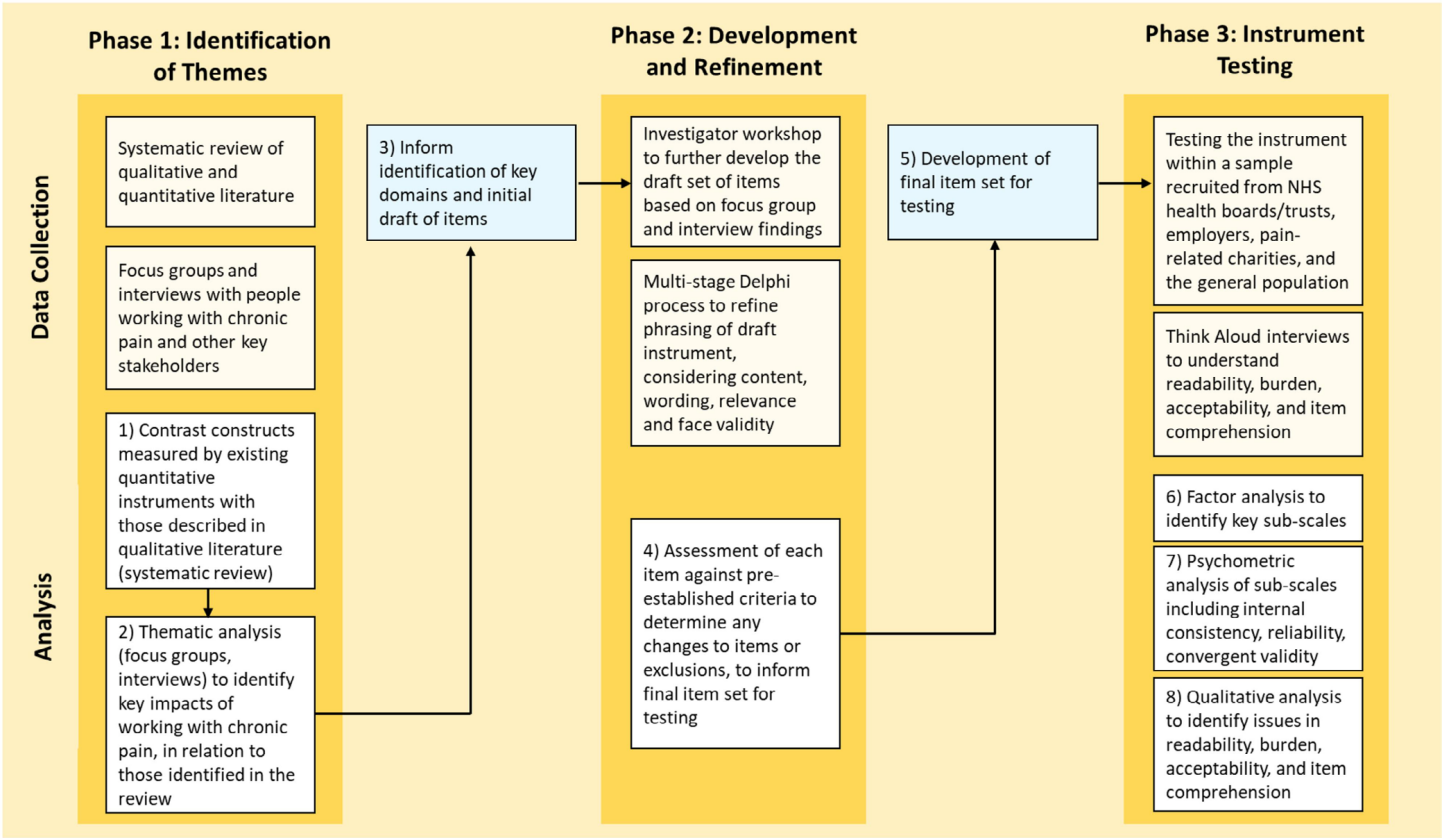
Overview of exploratory sequential mixed‐method design.

An overview of the exploratory sequential mixed‐methods design of this study (Creswell and Clark 2017) is provided in Figure 1. Across three phases of the study, qualitative and quantitative data were collected from people with lived experience of working with chronic pain and from other stakeholders, which were iteratively analysed to inform the development of the instrument. Within Phase 1, we identified key domains for inclusion, which were developed into items for the draft instrument within Phase 2. Within Phase 2, items were also iteratively refined before taking them forward for testing within Phase 3.

### 2.1 Phase 1: Identifying Key Domains to Include in WORC‐PAIN

People with chronic pain, employers, researchers, healthcare professionals (HCPs), and people working in third sector and policy settings, participated in deliberative focus groups (Rothwell et al. 2016) to broaden our understanding of lived experiences of working with chronic pain and how these related to the systematic review findings (Rothwell et al. 2016). Deliberative focus groups were carried out in order to orient participants to the findings of the systematic review (Stagg et al. 2022) and to provide an opportunity for interactive engagement with prepared videos which summarised results, for collaborative sense‐making (Rothwell et al. 2016). Individual interviews were carried out when it was not possible for a participant to attend a scheduled focus group.

People with experience of working with chronic pain (e.g., headache, rheumatic and musculoskeletal conditions, painful gynaecological conditions) were recruited via NHS clinical channels, relevant charities (e.g., the Migraine Trust, National Rheumatoid Arthritis Society), Patient & Public Involvement (PPI) groups associated with study investigators, and via studies within the Epidemiology Group at the University of Aberdeen in which participants had previously provided consent for contact about other studies. Participants were purposively sampled, prioritising diversity of clinical and occupational backgrounds, and we also aimed to include participants representing diverse sociodemographic characteristics across the lifecourse. Employers, researchers, HCPs, and people working in third sector and policy settings were identified and recruited via existing professional networks. Informed consent was obtained individually prior to each focus group or interview.

Ahead of the focus groups, we developed a series of videos which summarised findings from the systematic review to date on quantitatively‐assessed and qualitatively‐described impacts of chronic pain on work (Stagg et al. 2022). The videos were used to orient participants to the findings of the systematic review, provide the structure for the focus group discussions, and to ensure consistency in the educational component of the deliberative focus groups. Focus groups were held by MJS and EW on Microsoft Teams and audio recorded. MJS led the discussion, and EW acted as expert co‐moderator to field questions from participants about the topics for discussion and anything they were unclear about (Rothwell et al. 2016). Ahead of each online focus group/interview, participants were provided with links to the videos which included an introductory video and five thematic videos (e.g., workplace relationships, psychological factors) which were each 2.5–4 min long. Within these, MJS led participants through the results of the systematic review. The videos can be viewed via links provided within the ‘QUICK Deliberative Focus Group Videos’ document which can be accessed here: https://osf.io/hp4r2/files.

The videos were also played in the deliberative focus groups and they formed the basis for discussion (Rothwell et al. 2016). Following each video, participants discussed their thoughts on the findings and their experiences in relation to each video. Focus groups lasted approximately 90–120 min including a brief break if required and participants were given a £20 voucher to thank them for their time. Focus groups were transcribed verbatim and analysed thematically using an inductive and deductive approach (Braun and Clarke 2021; Proudfoot 2023), supported by NVivo coding software. MJS led the analysis of the focus groups, guided by analytic conversations (Locock et al. 2019) with the wider research team. This facilitated identification of overarching themes that were important to define the impacts of chronic pain on work that mattered to people and other stakeholders. Existing theory was used to enhance understanding, for example sociological and psychological understandings of self‐identity and personal value in relation to work, self‐efficacy, and models of job demand‐control and fear avoidance.

The focus group study was approved by the East of England—Essex Research Ethics Committee (reference: 22/EE/0095).

### 2.2 Phase 2: Drafting and Refining WORC‐PAIN to Ensure Face and Content Validity for People Working With Chronic Pain and Other Stakeholder Groups

#### 2.2.1 Drafting the Item Set and Workshops

Initial questions were drafted by MJS with input from LM based on the themes related to the impacts of chronic pain that were described in the Phase 1 qualitative focus groups/interviews. Items were drafted to reflect the range of impacts described within the focus groups, with consideration also given to the frequency that the impacts were mentioned. Some of our themes also informed the structure of how questions were asked. For example, participants often emphasised that they strongly disliked numerical scales for pain severity, so we avoided using such scales in the instrument. Consideration was given to the scope of each question in relation to experiences described in the focus groups and to the relevance of each item for different occupational groups. This ensured the drafted items subjectively reflected the impacts of chronic pain on work (face validity) and that they reflected the full scope of these impacts (content validity). Multiple items were drafted per each of the identified themes to facilitate subsequent discussion. A workshop with the research team was held following the analysis of focus group data and the initial drafting of item variants. Within this workshop, the research team reflected on the qualitative themes and discussed to reach consensus on grouping related items together and on reducing the overall number of items to a manageable number. During two subsequent meetings, the research team refined wording and the order of items to improve clarity and flow, and developed appropriate response options.

#### 2.2.2 Delphi Process

A multi‐stage Delphi process was conducted to further refine the phrasing of newly developed items, considering content, wording, relevance to diverse work settings, and face validity (Belton et al. 2019; McMillan et al. 2016). The aim of a Delphi process is to ultimately reach consensus from stakeholders on a research problem—in this case on the relevance and phrasing of items for the instrument. In contrast with the Phase 1 focus groups, the Delphi process involved discussing and voting on items, rather than establishing the primary themes for inclusion. The process also aimed to understand whether any additional constructs should be considered for inclusion. Participants included people with experience of working with chronic pain and individuals representing the same stakeholder groups as participated in the focus groups. People with experience of working with chronic pain were invited via relevant pain‐related charities, Patient & Public Involvement (PPI) groups associated with study investigators, and via studies within the Epidemiology Group at the University of Aberdeen in which participants had previously provided consent for contact about other studies. Stakeholders were approached about the study via relevant professional networks. Informed consent was obtained prior to participation.

Stage 1 of the Delphi process was undertaken in online Delphi focus groups held by MJS and SV on Microsoft Teams. Each draft item was discussed sequentially, with Delphi focus group participants proposing potential modifications to the instrument. Subsequently, all Delphi focus group participants were invited to a voting workshop where changes to items were proposed. Participants voted anonymously on whether the phrasing of each item and its response options were clear. We established decision rules for voting outcomes within the study protocol, prior to commencing the study. If fewer than 70% of participants agreed on an item, further revisions were made before the following phase by the research team, using suggestions presented in the voting workshop (Humphrey‐Murto et al. 2017).

During Stage 2, the revised instrument was sent to a larger panel of people with experience of working with chronic pain and stakeholder groups as part of an online survey. In the survey, participants were informed that we wanted to understand the relevance of each draft question in terms of how well it asked about the impact of chronic pain on work, and participants were then asked for each item, ‘How would you rate the relevance of each of the following questions?’ Ratings were given for each item on a 1–9 scale (not at all relevant—extremely relevant). We established decision rules for scores within the study protocol, prior to commencing the study. If a score of less than 7 was given, they were asked if (and how) the proposed question could be modified to improve its relevance. An item was taken forward if ≥ 70% of all participants scored it 7–9. An item was not included if ≥ 70% of participants scored it 1–3. All items not meeting these criteria could potentially be taken forward for evaluation in a second round of the survey.

The Delphi study was approved by the University of Aberdeen School of Medicine, Medical Sciences, and Nutrition Ethics Review Board (reference: SERB/2022/7/2417).

### 2.3 Phase 3: Testing the WORC‐PAIN Instrument (Validation and Scoring)

#### 2.3.1 Validation Survey

People with chronic pain currently in work from four recruitment settings were invited to complete the draft WORC‐PAIN instrument via an online questionnaire. Recruitment settings included: selected employers (e.g., Liverpool University Hospitals Foundation Trust, University of Aberdeen); four NHS health boards/trusts (including e.g., pain clinics, rheumatology, gynaecology, a Scottish registry of patients who provided consent to be contacted about relevant studies (SHARE) (McKinstry et al. 2017)); relevant charities (e.g., Crohn's & Colitis UK, National Axial Spondyloarthritis Society); and the general population. For the latter, participating general practices (*n* = 34) from 9 of 14 health boards in Scotland sent invitations to a random selection of registered patients (total invitations = 7775). A subsample of individuals who completed the baseline survey and who provided consent for further contact were invited to complete WORC‐PAIN again after two weeks to assess test–retest reliability.

Included within the online questionnaire were demographic, occupational and clinical questions for descriptive purposes (e.g., Widespread Pain Index (WPI) and Symptom Severity Scale (SSS) (Wolfe et al. 2010); Patient Health Questionnaire (PHQ)‐2 (Kroenke et al. 2003); general activity and sleep questions from the Brief Pain Inventory (BPI) (Cleeland 2021)). Information on job and industry were used for coding occupations according to the Standard Occupational Classification (SOC) (2020) system, using the Computer Assisted Structured Coding Tool (Cascot) to aid the coding process. The Work Productivity and Activity Impairment: General Health (WPAI:GH) (Reilly et al. 1993) questionnaire and ‘current work ability item’ from the Work Ability Index (WAI) (Ilmarinen 2007) were included for assessing convergent validity. Free‐text questions asked about: any missing constructs; any embarrassing, upsetting, or overly personal questions; and any other comments.

Items included within the draft WORC‐PAIN questionnaire represented diverse impacts of working with chronic pain and unique descriptive items for contextualising an individual's experience of chronic pain at work (e.g., whether they had disclosed their pain at work). Given the complexity and variation of the items, it was decided to analyse the responses by identifying relevant subscales. An exploratory factor analysis of items was therefore conducted to identify latent constructs within WORC‐PAIN (Goretzko et al. 2021). The factor analysis was conducted using a polychoric correlation matrix of the ordinal scores of Likert items focused on the impacts of working with chronic pain (e.g., not the descriptive contextual items). A principal axis factor extraction method was used, and factors were rotated using the oblique oblimin method. Factor loadings ≥ 0.45 were considered important and the number of factors to retain was determined by a multifaceted approach including consideration of eigenvalues > 1, a factor to item ratio of at least 1:3, and theoretical considerations including interpretability of the solutions (Goretzko et al. 2021; Tabachnick and Fidell 2007). The results of the factor analysis were used to inform refinements to the instrument, including the removal of items not contributing to the final factor structure. A series of research team meetings were held to discuss and reach consensus on the number of factors to retain based on the factor retention criteria, including clinical interpretability and utility.

Likert responses for items within each of the identified factors were transformed to a 0–6 scale to ensure equal weighting of items within the generation of subscales. Subscale scores were generated by summing items within each identified factor.

Missingness of data for each WORC‐PAIN item was assessed, as high levels of missing data could indicate issues of clarity or relevance. The distribution of responses was also considered because a high proportion of individuals selecting one end of each scale (i.e., ‘ceiling’ or ‘floor’ effects) or the same response option may indicate that responses do not reflect differences between participants. Following the description of item‐level missing data, the identification and definition of subscales and subsequent statistical analyses were conducted using complete cases. Each identified subscale sum score was tested for internal consistency using Cronbach's α. Spearman correlations were used to assess the convergent validity of the subscale sum scores with the WPAI:GH domains and WAI. Higher subscale scores (i.e., greater impact of working with chronic pain) were hypothesised to correspond with higher scores on the WPAI:GH domains (i.e., greater productivity and activity impairment) and with lower scores on the WAI (i.e., less able to work compared with lifetime best) (Abma et al. 2016). Test–retest reliability was assessed using the intraclass correlation coefficient (ICC) to test score agreement between each subscale sum score at baseline and at retest. ‘Good’ test–retest reliability was defined as correlations > 0.75 (Koo and Li 2016).

Statistical analyses were conducted using STATA/SE V17.0.

#### 2.3.2 Think Aloud Interviews

A concurrent online Think Aloud study was conducted alongside the validation survey. In the context of developing a new instrument, Think Aloud interviews can be used to understand its readability, responder burden, acceptability, and individuals' comprehension of items (Drennan 2003; Padilla and Leighton 2017). MJS led the Think Aloud interviews. People with experience of working with chronic pain invited from the PPI and existing study networks described above were asked to ‘complete’ the draft WORC‐PAIN instrument out loud, by reading through the questions and answers. They were also asked to verbalise their thoughts while completing it. This aimed to facilitate an understanding of how they processed and understood the draft WORC‐PAIN instrument in real‐time (Padilla and Leighton 2017). A subsequent reflective phase allowed for deliberative questions for ‘elaboration or ‘explanation’ (e.g., for having considered multiple options for a question) (Padilla and Leighton 2017). During the second phase we also asked about general perceptions of the draft WORC‐PAIN instrument including its acceptability, feasibility and responder burden. Data were analysed based on existing think aloud coding frameworks to identify any issues (Aujla et al. 2020; French et al. 2007; Van Oort et al. 2011).

Results from the Think Aloud study, factor analysis, and survey free text responses were considered amongst co‐investigators and discussed with patient partners to inform final minor refinements to the WORC‐PAIN questionnaire.

The validation survey and Think Aloud study were approved by the East of England—Essex Research Ethics Committee (reference: 22/EE/0095).

Patient research partners with experience of working with chronic pain were involved throughout the study. A core group of three research partners was involved in the development and delivery of each study phase, and a wider group of individuals was involved in testing and feeding back on study processes. For example, research partners were involved in the development of the videos presented within the Phase 1 focus groups, piloting the Phase 2 Delphi process, refining revisions to the instrument within the Delphi process, and finalising question order and instructions following the final survey (Phase 3).

## 3 Results

### 3.1 Phase 1: Identifying Key Domains to Include in WORC‐PAIN

Thirty‐two people with experience of living and working with chronic pain participated in six focus groups and three interviews, and 18 stakeholders participated in four focus groups. Descriptive characteristics of participants are provided in Table 1 within S1.

**FIGURE 2:**
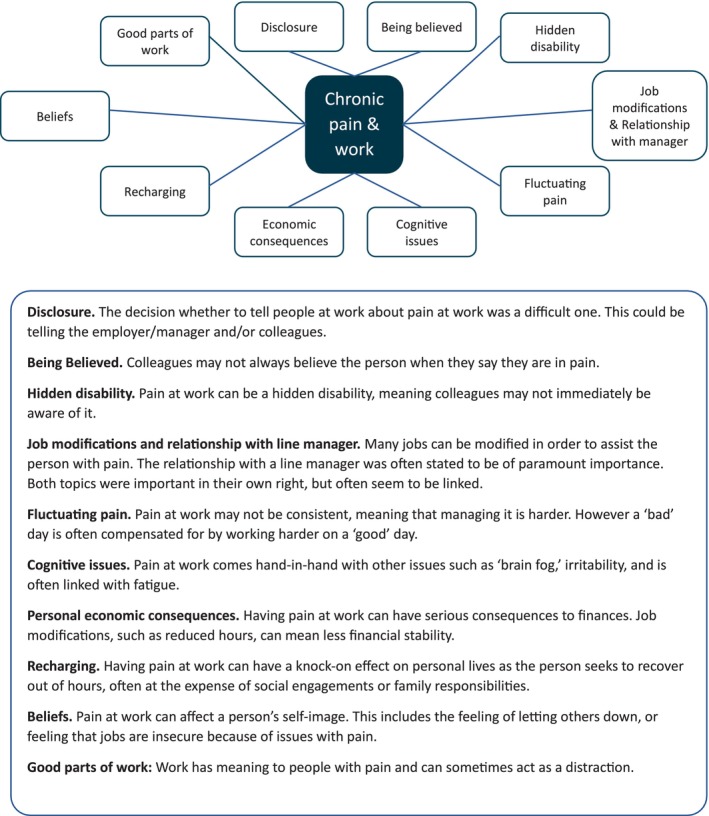
Focus group findings: Key topics relating to the impact of chronic pain on work.

Findings from the systematic review resonated with participants. However, impacts related to the quantity and quality of work, which were the focus of quantitative tools to date, were thought to represent only one aspect of the multifaceted impacts of chronic pain on work. Ten key topics were generated from the thematic analysis and used as the basis for informing item development. These are illustrated and described in Figure 2. Within the focus groups, participants described, for example, the impacts of working with chronic pain on their personal finances and financial stability, varied cognitive impacts like reduced concentration and increased irritability, and wider impacts relating to the need to recharge after work, which in turn reduced their social engagement with friends and family. Participants also described positive aspects of work, for example how positive line manager support could enable job modifications, and how work itself could serve as a distraction from the experience of pain.

### 3.2 Phase 2: Drafting and Refining WORC‐PAIN to Ensure Face and Content Validity for People Working With Chronic Pain and Other Stakeholder Groups

#### 3.2.1 Drafting the Item Set and Workshops

Using findings from the focus groups, an initial set of items was drafted based on identified themes. Items were refined in terms of their wording and order during a subsequent research team workshop and meetings. Eighty‐four item variants were initially discussed by the team and 61 were taken forward for refinement within the subsequent Delphi process.

#### 3.2.2 Delphi Process

Twelve people with experience of working with chronic pain participated in three Delphi focus groups and three stakeholders participated in one Delphi focus group within Stage 1 of the Delphi process. Characteristics of the Delphi focus group participants are provided in Table 2 within S1. Several minor changes were made to the draft WORC‐PAIN questionnaire following the Delphi focus groups (e.g., adding a ‘skip’ option for people who had not disclosed their pain at work and therefore could not answer a subsequent question about who they had disclosed to; splitting a question about job modifications into physical‐ and schedule‐based modifications). All items, including any changes, were proposed to participants during the subsequent Stage 1 workshop. Eight Delphi focus group participants voted on items within the workshop. All but one item passed the voting threshold. This item was only relevant for people who had not disclosed their pain at work and the instructions on who should complete it (i.e., those who had not disclosed) were unclear. This was revised prior to taking it forward to the Stage 2 Delphi survey.

55 participants completed the Stage 2 Delphi survey. This included 19 people with chronic pain and 36 stakeholders (see Table 3 within S1 for descriptive characteristics). All items in the draft WORC‐PAIN questionnaire passed the required threshold within the first round of the Stage 2 survey, and a second round of the survey was therefore not required.

### 3.3 Phase 3: Testing the WORC‐PAIN Instrument (Validation and Scoring)

#### 3.3.1 Validation Survey

2120 individuals completed initial eligibility questions within the online survey, confirming whether they experienced chronic pain and were currently in work. Of these, 1044 (49.2%) eligible individuals provided informed consent. Of those who provided consent, 919 (88.0%) provided any data within the WORC‐PAIN questionnaire and therefore comprised the sample for the descriptive WORC‐PAIN item analysis. Of the sample, 281 (30.6%) individuals were recruited via participating employers, 198 (21.5%) via participating health boards/trusts, 210 (22.9%) via relevant charities, and 230 (25.0%) from the general population. Sample characteristics are provided in Table 1. Overall, most participants were women (80%) and the sample represented an older working population (median age 50, IQR 39–56). 12% had more than one job, and most participants (86%) were currently working for an employer. Within their main job, 18% were working non‐standard working routines, 40% worked a shift pattern, and 14% worked night shifts at least some of the time. All nine SOC codes were represented in the sample (Table 1). Professional (35.9%), administrative and secretarial (15.8%), and associate professional (13.1%) occupations were the most highly represented. Most participants experienced pain in multiple areas of the body (WPI median score 5; IQR 3–8) of moderately high severity (SSS median score 8; IQR 6–10). Participants' general activities and sleep were also affected by their pain (BPI median (IQR) scores 6 (4–8) and 7 (4–8), respectively).

**TABLE 1:** Characteristics of the validation sample, *n* = 919.

Demographic characteristics	*N* (%)
Age	
18–29	78 (8.5)
30–39	153 (16.7)
40–49	211 (23.0)
50–59	314 (34.2)
60+	141 (15.3)
Missing	22 (2.4)
Gender	
Man	157 (17.1)
Woman	738 (80.3)
Non‐binary	8 (0.9)
Prefer not to say	5 (0.5)
Missing	11 (1.2)
Ethnicity	
Asian	10 (1.1)
Black	5 (0.5)
Mixed or multiple ethnic groups	13 (1.4)
White	884 (96.2)
Another ethnic group	3 (0.3)
Prefer not to say	4 (0.4)
Education	
No formal qualifications	11 (1.2)
Secondary	99 (10.8)
College	225 (24.5)
Professional	158 (17.2)
Undergraduate	231 (25.1)
Postgraduate	195 (21.2)
Work characteristics	
Number of jobs	
One job	806 (87.7)
> 1 job	106 (11.5)
Missing	7 (0.8)
Standard occupational classifications, main job	
Major group 1 (managers, directors, senior officials)	63 (6.9)
Major group 2 (professional occupations)	330 (35.9)
Major group 3 (associate professional occupations)	120 (13.1)
Major group 4 (administrative and secretarial occupations)	145 (15.8)
Major group 5 (skilled trades occupations)	32 (3.5)
Major group 6 (caring, leisure and other service occupations)	110 (12.0)
Major group 7 (sales and customer service occupations)	46 (5.0)
Major group 8 (process, plant and machine operatives)	22 (2.4)
Major group 9 (elementary occupations)	30 (3.2)
Volunteer	9 (1.0)
Missing	12 (1.3)
Work status, main job	
Employed	793 (86.3)
Employed, off sick	30 (3.3)
Self‐employed	52 (5.7)
Self‐employed, off sick	2 (0.2)
Voluntary work	18 (2.0)
Retired, some paid work	12 (1.3)
Retired, some voluntary work	9 (1.0)
Missing	3 (0.3)
Work schedule, main job	
Regular (*roughly same hrs every week*)	749 (81.5)
Rotating (e.g., *day shift some days, night shift other days*)	66 (7.2)
Irregular (*unpredictable hrs controlled by situations or workload*)	83 (9.0)
Contract work (*working as and when work is available*)	18 (2.0)
Missing	3 (0.3)
Shift work, frequency	
Never	540 (58.8)
Seldom	87 (9.5)
Sometimes	69 (7.5)
Very often	44 (4.8)
Always	168 (18.3)
Missing	11 (1.2)
Night work, frequency	
Never	750 (81.6)
Seldom	39 (4.2)
Sometimes	30 (3.3)
Very often	22 (2.4)
Always	40 (4.4)
Missing	38 (4.1)
Driving for work, frequency	
Never	589 (64.1)
Seldom	74 (8.1)
Sometimes	81 (8.8)
Very often	51 (5.6)
Always	100 (10.9)
Missing	24 (2.6)
Main earner in household	
Yes	532 (57.9)
Missing	2 (0.2)
Total contracted hours per week (Median, IQR)	35 (25–38)
NA *n* (%)	62 (6.8)
Missing *n* (%)	3 (< 0.01)
Average hours worked per week (Median, IQR)	37 (28–40)
Missing *n* (%)	30 (3.3)
Work productivity and activity impairment (Median, IQR)	
Absenteeism *(% of working hrs)*	0 (0,14)
Presenteeism (%)	40 (20,60)
(*100 = completely prevented from working*)	
Overall work impairment (%)	40 (20,70)
Non‐work activity impairment (%)	60 (40,80)
(*100 = completely prevented from doing activities*)	
Work ability index (0–10) (Median, IQR)	6 (5,8)
(*0 = completely unable to work; 10 = work ability at its best compared to lifetime best*)	
**Clinical measures**	**Median, IQR**
Widespread pain index (0–19)	5 (3,8)
Symptom severity scale (0–12)	8 (6,10)
PHQ2 (0–6)	2 (1,4)
Brief Pain Inventory	
General activity (0–10)	6 (4,8)
Sleep (0–10)	7 (4,8)

713 (77.6%) participants completed all WORC‐PAIN items and therefore comprised the sample for factor analysis. Participants included in the factor analysis (*n* = 713) did not differ from those not included (*n* = 206) in terms of gender (*χ* ^2^(3908) = 2.99, *p* = 0.39), age (*χ* ^2^(4897) = 7.58, *p* = 0.12), level of completed education (*χ* ^2^(5919) = 2.68, *p* = 0.75), working status (*χ* ^2^(6916) = 7.34, *p* = 0.29), and SOC code (*χ* ^2^(9907) = 3.97, *p* = 0.91). Thirty‐five items which had a clear positive/negative valence structure were entered into the factor analysis. Five items about disclosure, and one each focused on past career changes (e.g., reduced hours, changed jobs), perceived compensation for pain (activity adjustment), and perception of self were excluded from the factor analysis a priori as descriptive/contextual items. Within a series of meetings, the research team discussed possible factor solutions based on the analysis—in particular, two‐ and three‐ factor models. The three‐factor model was considered superior in terms of its clinical interpretability given its separation of proximal and distal work‐related impacts which generally collapsed into one factor within the two‐factor solution (i.e., the two‐factor solution solely included a ‘work and wider life impacts’ factor and a ‘facilitators’ factor). This separation of concepts was considered important for identifying distinctions in the ways work‐related impacts can affect individuals (e.g., at work or in their wider life) and would have implications for identifying relevant and targeted solutions. Three factors were therefore retained and 77% of the variance in the factor model was accounted for by these domains. 24 items contributed to these domains with factor loadings ≥ 0.45. The scree plot of factor eigenvalues is provided in Figure 1 within S1, and tables of factor loadings and factor correlations are provided in Tables 4 and 5 within S1, respectively.

The three identified factors grouped together questions that related to the following domains:

- Domain 1: wider impacts of working with chronic pain (‘wider impacts’);
- Domain 2: day to day impacts of working with chronic pain (‘day to day impacts’); and
- Domain 3: individual and job‐based facilitators to support working with chronic pain (‘facilitators’).

**FIGURE 3:**
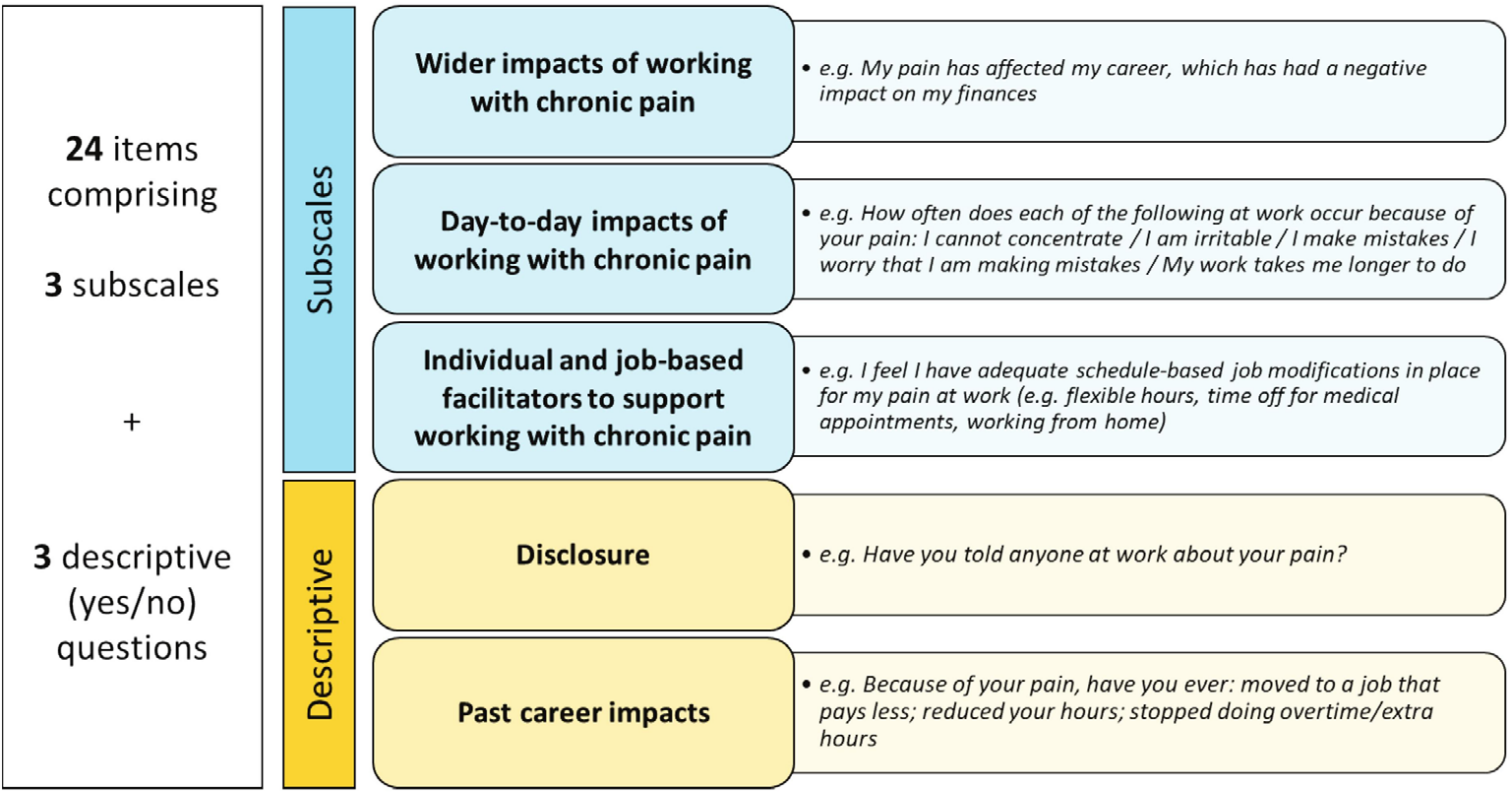
Final WORC‐PAIN structure and example items.

These domains are illustrated, along with example items, within Figure 3. For example, the ‘wider impacts’ domain includes the item, ‘My pain has affected my career, which has had a negative impact on my finances’; the ‘day to day impacts’ domain includes the items, ‘How often does each of the following at work occur because of your pain: I cannot concentrate; I am irritable; I make mistakes; I worry that I am making mistakes; My work takes me longer to do’; and the ‘facilitators’ domain includes the item, ‘I feel I have adequate schedule‐based job modifications in place for my pain at work (e.g., flexible hours, time off for medical appointments, working from home).’ One item within the ‘facilitators’ domain also loaded onto ‘wider impacts’. However it was retained within the ‘facilitators’ domain as it loaded more strongly onto this factor (0.48 vs. −0.45) and was also conceptualised as a positive facilitator to work by focus group participants. Eleven items were excluded from WORC‐PAIN subscales due to low loadings within the selected factor structure. However, all items included in the draft WORC‐PAIN questionnaire were developed and informed with significant public and stakeholder involvement and deemed important. Items not taken forward for the main WORC‐PAIN questionnaire were therefore included in a supplementary sheet. These additional items may be particularly useful for facilitating more detailed, constructive conversations about working with chronic pain in a clinical setting for example, or in specific research settings where one of these domains may be of particular research interest (e.g., reasons for not disclosing pain at work, effects of medication, feelings of comparison with others).

All items comprising WORC‐PAIN subscales were rescaled to a 0–6 scale to ensure equal weightings of items within each subscale, that is, to prevent specific items from contributing more or less to a given scale based on their response options. Sum scores were then generated for each domain to ensure ease of computing scores in diverse research and clinical settings (Hill et al. 2016). Domain 1 (‘wider impacts’) comprised 10 items with a possible score 0–60; Domain 2 (‘day‐to‐day impacts’) comprised 10 items with a possible score 0–60; and Domain 3 (‘Facilitators’) comprised 4 items with a possible score 0–24. Items are scored so that a higher score reflects a greater negative impact/negative experience of working with chronic pain. Therefore a higher score on Domain 1 would indicate greater negative impacts of working with chronic pain on an individual's wider life and expectations for the future, while a higher score on Domain 3 would indicate that the individual has fewer facilitators in place that support their work. Two items within Domain 3 which are positively framed are reverse coded to achieve this. Detailed scoring instructions for each item and subscale are included within the full questionnaire: https://osf.io/hp4r2/files.

All WORC‐PAIN items had < 10% missing data and none contained a response option where the majority of the sample had selected it. This indicated that available response options within subscales captured diverse experiences.

Cronbach's *α* for each domain indicated that each subscale could be considered as a scale (Cronbach's *α* of 0.89, 0.87, and 0.60 for ‘wider impacts’, ‘day‐to‐day impacts’, and ‘facilitators’, respectively).

Spearman correlations between the WORC‐PAIN subscales ‘wider impacts’ and ‘day‐to‐day impacts’ and the WAI, and presenteeism and overall work impairment within the WPAI were moderate‐high (Table 2). Correlations between these subscales and absenteeism (WPAI) were moderate (Table 2). Correlations between the WORC‐PAIN ‘facilitators’ subscale and each of the WPAI measures and the WAI were weak. The direction of correlation was as hypothesised in all cases that is, higher scores on the WORC‐PAIN subscales corresponded with higher scores on the WPAI constructs and with lower scores on the WAI (Table 2).

**TABLE 2:** Convergent validity; correlations between WORC‐PAIN subscales and the WPAI:GH constructs and the WAI.

Working with chronic pain domain	Correlation (ρ, 95% CI)
Wider impacts of working with chronic pain	
WPAI—absenteeism	0.40 (0.34, 0.46)
WPAI—presenteeism	0.59 (0.54, 0.64)
WPAI—overall work impairment	0.60 (0.55, 0.64)
WAI	−0.52 (−0.56, −0.46)
Day‐to‐day impacts of working with chronic pain	
WPAI—absenteeism	0.41 (0.35, 0.47)
WPAI—presenteeism	0.63 (0.58, 0.67)
WPAI—overall work impairment	0.64 (0.60, 0.68)
WAI	−0.51 (−0.56, −0.46)
Individual and job‐based facilitators to support working with chronic pain	
WPAI—absenteeism	0.12 (0.05, 0.19)
WPAI—presenteeism	0.14 (0.07, 0.21)
WPAI—overall work impairment	0.12 (0.04, 0.19)
WAI	−0.17 (−0.24, −0.11)

Abbreviations: WAI, Work Ability Index; WPAI, Work Productivity and Activity Impairment Questionnaire.

73 participants who provided consent for future contact were invited to complete the retest questionnaire. Invitations were sent two weeks after completion of the initial survey. 53 participants provided complete retest data, with a median completion time of 20 days after the first questionnaire (IQR: 16–24 days). ICCs between each subscale were good at 0.92 (95% CI: 0.86, 0.95) for ‘wider impacts’, 0.89 (95% CI: 0.81, 0.93) for ‘day‐to‐day impacts’, and 0.82 (95% CI: 0.70, 0.89) for ‘facilitators’.

#### 3.3.2 Think Aloud Interviews

Nine individuals with chronic pain participated in the Think Aloud study (seven women; two participants < 30 years old, two 30–49, four 50–59 (1 missing); seven in part‐time work; four self‐employed). The Think Aloud process and free text responses to the survey were analysed to determine whether there were any issues of clarity or with interpretation of the items, revealing that items and response options were interpreted as intended.

Several workshops were held with the research team to agree the final instrument taking account of all the findings. In addition to the 24 items taken forward from the factor analysis, three descriptive questions from the tested instrument were included in the final version to provide important contextual information critical to interpreting the impacts of working with chronic pain that one might experience. These included items about whether they had disclosed their pain at work, to whom they had disclosed, and a question about past career impacts/changes due to chronic pain. The final instrument was then compiled and reviewed in a meeting with patient partners to determine the completion time (5–10 min), and to agree the final question order and instructions. A summary of the final instrument structure, domains and example items is included in Figure 3.

The full *Working with Chronic Pain: Assessment of Impacts* (WORC‐PAIN) questionnaire, along with supplementary item sheet, is available for use at: https://osf.io/hp4r2/files.

## 4 Discussion

We have developed a valid and reliable multidimensional instrument which captures the impacts of working with chronic pain as defined by people with chronic pain. The final instrument is based on input from over 100 people with experience of working with chronic pain and other key stakeholders including healthcare professionals, employers, researchers, and people working in the third sector and policy settings. The WORC‐PAIN questionnaire is relevant and understandable to individuals with pain in diverse clinical and occupational contexts, and it measures factors that are important to people working with chronic pain. It offers greater insight into the difficulties of working with chronic pain, the day‐to‐day effects and what types of support or intervention might better enable people with chronic pain to work.

The QUICK Study comprised a methodologically robust, exploratory sequential mixed methods design to ensure that the WORC‐PAIN questionnaire captures information on domains important to people working with chronic pain, and to ensure that it is a valid and reliable tool in doing so. We aimed to include people from diverse clinical and occupational contexts. While some participants were already linked to formal networks in some way, many were not. For example, our study invitation for the Phase 3 survey was sent to all staff within NHS Grampian which included for example, cleaners, cooks, grounds staff, administrative staff, and healthcare professionals. Additionally, our general population sample was invited from a random sample of people registered with each participating general practice, which also allowed us to recruit a range of people representing diverse occupational characteristics. We have achieved occupational diversity as indicated by for example, representation of a range of SOC codes, shift patterns, and employment contexts within the Phase 3 validation survey. Our final testing of the WORC‐PAIN questionnaire demonstrated that the tool is relevant in these different settings, as indicated by the minimal missing data across the WORC‐PAIN items and the range of responses selected in response to each item. However, within the context of aiming for clinical and occupational diversity, we acknowledge that capturing more individual diversity would have been beneficial. Our samples comprised mostly women, most participants were white, and many had completed undergraduate or postgraduate degrees. This reflects challenges in research generally and in going forward we will make a concerted effort to understand how to best use the instrument in more diverse populations.

Across the three WORC‐PAIN subscales, the third (‘individual and job‐based facilitators to support working with chronic pain’) had the weakest internal consistency (*α* = 0.60). However, internal consistency is affected by the number of items within a scale, and it is therefore unsurprising that this subscale, which is made up of only four items, had a lower internal consistency rating than the other two subscales which are each made up of ten. Future testing could identify whether the inclusion of an additional, conceptually similar item could improve this, but this would need to be weighed up against the potential for redundancy of the information captured by it. Additionally, this subscale is conceptualised to likely be formative, rather than reflective, in that the specific facilitators assessed would be theorised to give rise to feeling supported or positive about working with chronic pain. Measures of internal consistency may be less appropriate for formative constructs (Myszkowski et al. 2019). This subscale also had the weakest correlations with the WPAI:GH constructs and WAI when testing for convergent validity. However, the weaker correlations between this subscale and the reference instruments, coupled with the fact that the two WORC‐PAIN subscales assessing ‘impacts’ had the strongest correlations with ‘overall work impairment’ construct (within the WPAI:GH), strengthen the argument for the construct validity of the subscales as the reference instruments used are not ‘gold standards’ for work impairment and they fail to capture what matters for people with chronic pain.

Each subscale reflected good test–retest reliability. It is possible that individuals' symptom(s) fluctuated during the assessment time period. However, most questions in WORC‐PAIN are phrased so that they ask about participants' experiences overall or generally and we would therefore expect within‐person symptom fluctuation to have little effect over this time period.

Throughout the development of the WORC‐PAIN instrument, we navigated the sharing and inclusion of diverse perspectives from people with experience of working with chronic pain, and from other stakeholders. This was undertaken through several different qualitative and quantitative methods which required ongoing elaboration, synthesis, and integration as no singular perspective nor methodology had the final say in what was included. This understandably led to some tensions and challenges given there were at times different priorities and expectations. We approached these challenges using the concept of ‘analytic conversations’ (Locock et al. 2019), which, as Hollick et al. outline, ‘are more ethos than method, centred on shared learning, mutual care, and responsible attention to different perspectives. They represent an ethos of learning together: ongoing discussions shape decisions, differing perspectives are welcomed even if uncomfortable, and with an ethic of responsibility to attend to these views*’* (Hollick et al. 2024). The exploratory sequential mixed‐methods design allowed time and space for perspectives to be reflected upon at the beginning of each study phase, and we used the concept of analytic conversations to ensure there was transparent and inclusive decision making at each stage.

We included people with diverse working arrangements in the development and testing of the WORC‐PAIN questionnaire. This has ensured that questions and response options can be meaningfully interpreted, regardless of an individual's working context. While this instrument was developed in the UK, many of the impacts it describes are likely to be relevant in other countries, and indeed our systematic review that informed our qualitative work synthesised impacts of chronic pain on work that have been experienced in other countries, for example, Australia, Canada, France, Sweden (Stagg et al. 2022). In different settings and healthcare systems, cultural and social contexts will inevitably shape how these impacts are experienced and the solutions proposed, yet the impacts may still resonate. This is also likely to be the case across diverse occupational contexts (e.g., professional vs. skilled trades or process operative roles) and within more ethnically diverse populations. Exploring this in diverse contexts would be a valuable area for future research and cross‐cultural validation could ensure the impacts assessed retain conceptual equivalence (Cruchinho et al. 2024). Future studies should aim to capture and report on the cultural, social, and occupational contexts to facilitate an understanding of context‐dependent differences that shape experiences of these impacts.

WORC‐PAIN can be used to quantify relevant and important impacts of chronic pain at an individual level, within a particular group, and at a population level. Furthermore, it could be used to quantify changes in personally‐meaningful impacts within clinical, occupational, and public health research settings resulting from for example, changes to treatment, a workplace intervention, or a change in policy. To date, work outcomes that have been included in clinical trials do not capture impacts that are of prime importance to individuals working with chronic pain (e.g., Sowah et al. 2018; Ter Wee et al. 2012). We have demonstrated that productivity, as captured by existing instruments, is a small part of the picture and WORC‐PAIN offers the potential to demonstrate change in important impacts resulting from novel interventions for pain‐related conditions.

The WORC‐PAIN questionnaire could also be utilised as a tool for identifying solutions. Interventions which support and facilitate ‘good work’ for an individual (Black 2008) have the potential to act as ‘facilitators to support working with chronic pain’, thereby reducing ‘day‐to‐day impacts of working with chronic pain’ and ultimately, ‘wider impacts of working with chronic pain’. WORC‐PAIN may therefore be able to quantify the importance of ‘good work’ for people working with chronic pain and could be used as a tool both for promoting and protecting health (Sorensen et al. 2016). Future work could aim to assess responsiveness to change. Minimal clinically important differences (MCID) based on the implementation of targeted vocational interventions could also be considered; however, defining these is likely to be challenging given the impacts experienced will depend on personal and occupational contexts. Given the significant role of patient and public involvement in the development of WORC‐PAIN, patient and public perspectives should also largely guide the development of MCIDs by ensuring differences or changes on WORC‐PAIN subscales relate to personally relevant and subjective levels of improvements experienced within known, effective interventions (McGlothlin and Lewis 2014).

## 5 Conclusions

WORC‐PAIN has been successfully developed with extensive input from people with chronic pain and relevant stakeholders to quantify impacts of working with chronic pain at a population level. The multidimensional impacts captured by the WORC‐PAIN subscales reflect the importance of the workplace environment and relationships, consequences on perceptions of work ability, expectations about the future, personal economic impacts, and wider interactions between living and working with chronic pain. It has undergone psychometric testing and has strong test–retest reliability, internal consistency, and demonstrates construct validity with reference work ability and impairment measures. Future studies will examine responsiveness to change and the potential for mapping identified impacts to interventions.

## Author Contributions

**R. Stuart Anderson:** project administration. **Nicola Goodson:** conceptualisation, resources. **Rosemary Hollick:** conceptualisation, methodology, formal analysis, visualisation, writing – review and editing. **Gary J. Macfarlane:** conceptualisation, supervision, writing – review and editing. **Ira Madan:** conceptualisation, methodology, writing – review and editing. **LaKrista Morton:** conceptualisation, methodology, formal analysis, visualisation, writing – original draft, writing – review and editing, and project administration. **Martin J. Stevens:** methodology, investigation, formal analysis, writing – review and editing, visualisation. **M. M. Suzanne Verstappen:** conceptualisation, methodology, investigation, writing – review and editing. **Elaine Wainwright:** investigation, formal analysis, writing – review and editing. **Karen Walker‐Bone:** conceptualisation, methodology, writing – review and editing.

Additional affiliations for M.M.S.V. include Arthritis UK Centre for Epidemiology, Centre for Musculoskeletal Research, Division of Musculoskeletal and Dermatological Sciences, School of Biological Sciences, Faculty of Biology, Medicine and Health, The University of Manchester, Manchester, UK; NIHR Manchester Biomedical Research Centre, Manchester University NHS Foundation Trust, Manchester Academic Health Science Centre, Manchester, UK.

## Funding

The work presented in this manuscript was funded by the Medical Research Council (Grant MR/V020676/1) and supported by the Arthritis UK/Medical Research Council Centre for Musculoskeletal Health and Work (Grant 22090).

## Disclosure

*Use of artificial intelligence*: None used.

## Conflicts of Interest

The authors declare no conflicts of interest.

## Supporting information


**Figure S1:** Eigenvalues of factors used to inform factor retention.
**Table S1:** Characteristics of focus group participants.
**Table S2:** Characteristics of Delphi focus group participants (Stage 1).
**Table S3:** Characteristics of Delphi survey participants (Stage 2).
**Table S4:** Extracted factor loadings.
**Table S5:** Extracted factor correlations.

**Data S1:** WORC‐PAIN_GRAMMS checklist.
