# Supplementary material for: Development and Validation of the ‘Working With Chronic Pain: Assessment of Impacts’ (WORC‐PAIN) Questionnaire for People in Paid Work With Chronic Pain
Source: Eur J Pain. 2026 Jul 29;30(7):e70337. doi: 10.1002/ejp.70337 (PMC13420923; doi:10.1002/ejp.70337)
Supplement: Supplementary file 1 — Figure S1: Eigenvalues of factors used to inform factor retention. Table S1: Characteristics of focus group participants. Table S2: Characteristics of Delphi focus group participants (Stage 1). Table S3: Characteristics of Delphi survey participants (Stage 2). Table S4: Extracted factor loadings. Table S5: Extracted factor correlations. [file EJP-30-0-s002.docx]

# Supplementary File Table 1: Characteristics of focus group participants

| **People with chronic pain, demographic characteristics** (n=32) | **N (%)** |
| --- | --- |
| **Age**  18-29  30-39  40-49  50-59  60+  *Missing* | 3 (9.4)  2 (6.3)  6 (18.8)  14 (43.8)  7 (21.9)  0 (0) |
| **Gender**  Man  Woman  Non-binary  *Missing* | 6 (18.8)  26 (81.3)  0 (0)  0 (0) |
| **Ethnicity**  Asian  Black  Mixed or multiple ethnic groups  White  Another ethnic group  Prefer not to say  *Missing* | 0 (0)  1 (3.1)  1 (3.1)  30 (93.8)  0 (0)  0 (0)  0 (0) |
| **Education**  No formal qualifications  Secondary  College/vocational qualification  Professional qualification  Undergraduate degree  Postgraduate degree | 0 (0)  1 (3.1)  4 (12.5)  8 (25.0)  9 (28.3)  10 (31.3) |
| **Work status, main job**  Full-time  Part-time  Unpaid work (e.g. carer)  Unemployed and seeking work  Unemployed because of ill health/disability (and not seeking work)  Retired  Retired early because of ill health/disability  Student  *Missing* | 13 (40.6)  10 (31.3)  1 (3.1)  2 (6.3)  1 (3.1)  1 (3.1)  3 (9.4)  1 (3.1)  0 (0) |
| **Working context, main job**  Zero-hours contract  Temporary contract  Self-employed | 1 (3.1)  4 (12.5)  4 (12.5) |
| **Stakeholders** (n=18) | **N (%)** |
| **Occupational group**  Employer  Healthcare professional  Researcher / academic  Working in policy setting  Other  Missing | 2 (11.1)  10 (55.6)  3 (16.7)  0 (0)  3 (16.7)  0 (0) |

# Supplementary File Table 2: Characteristics of Delphi focus group participants (Stage 1)

| **People with chronic pain, demographic characteristics** (n=12) | **N (%)** |
| --- | --- |
| **Age**  18-29  30-39  40-49  50-59  60+  *Missing* | 0 (0)  5 (41.7)  1 (8.3)  3 (25.0)  2 (16.7)  1 (8.3) |
| **Gender**  Man  Woman  Non-binary | 2 (16.7)  10 (83.3)  0 (0) |
| **Ethnicity**  Asian  Black  Mixed or multiple ethnic groups  White  Another ethnic group | 0 (0)  1 (8.3)  0 (0)  11 (91.7)  0 (0) |
| **Education**  No formal qualifications  Secondary  College/vocational qualification  Professional qualification  Undergraduate degree  Postgraduate degree | 0 (0)  1 (8.3)  0 (0)  1 (8.3)  6 (50.0)  4 (33.3) |
| **Work status, main job**  Full-time  Part-time  Unpaid work (e.g. carer)  Unemployed and seeking work  Unemployed because of ill health/disability (and not seeking work)  Retired  Retired early because of ill health/disability  Student | 6 (50.0)  2 (16.7)  0 (0)  1 (8.3)  0 (0)  2 (16.7)  1 (8.3)  0 (0) |
| **Stakeholders** (n=3) | **N (%)** |
| **Occupational group**  Employer  Healthcare professional  Researcher / academic  Working in policy setting  Other | 0 (0)  0 (0)  3 (100)  0 (0)  0 (0) |

# Supplementary File Table 3: Characteristics of Delphi survey participants (Stage 2)

| **Demographic characteristics, people with chronic pain** (n=19) | **N (%)** |
| --- | --- |
| **Age**  18-29  30-39  40-49  50-59  60+  *Missing* | 1 (5.3)  0 (0)  1 (5.3)  7 (36.8)  10 (52.6)  *0 (0)* |
| **Gender**  Man  Woman  Non-binary | 2 (10.5)  17 (89.5)  0 (0) |
| **Ethnicity**  Asian  Black  Mixed or multiple ethnic groups  White  Another ethnic group  Prefer not to say | 0 (0)  0 (0)  0 (0)  19 (100)  0 (0)  0 (0) |
| **Education**  No formal qualifications  Secondary  College/vocational qualification  Professional qualification  Undergraduate degree  Postgraduate degree | 0 (0)  1 (5.3)  3 (15.8)  2 (10.5)  9 (47.4)  4 (21.1) |
| **Work status, main job**  Full-time  Part-time  Unpaid work (e.g. carer)  Unemployed and seeking work  Unemployed because of ill health/disability (and not seeking work)  Retired  Retired early because of ill health/disability  Student | 4 (21.1)  3 (15.8)  0 (0)  0 (0)  3 (15.8)  2 (10.5)  6 (31.6)  1 (5.3) |
| **Occupational group, stakeholders** (n=36) | **N (%)** |
| **Occupational group** (note that 7 stakeholders had 2 occupational roles; *n* therefore sums to 43)  Employer  Healthcare professional  Researcher / academic  Working in policy setting  Third sector | 2 (5.6)  18 (50.0)  14 (38.9)  2 (5.6)  7 (19.4) |

# Supplementary File Table 4: Extracted factor loadings

| **Item (summary)** | **Factor 1** | **Factor 2** | **Factor 3** |
| --- | --- | --- | --- |
| Financial impact | **0.46** | 0.23 | 0.10 |
| Future expectations:  Stop work completely  Reduce hours | **0.60**  **0.45** | -0.04  0.10 | 0.14  0.17 |
| Fear of losing job | **0.56** | 0.27 | 0.10 |
| Perception of not being able to find other work | **0.60** | 0.08 | 0.01 |
| Working with chronic pain’s impact on:  Self-care  Housework  Time with family  Seeing friends  Doing leisure activities | **0.56**  **0.68**  **0.81**  **0.85**  **0.81** | 0.25  0.21  0.06  0.07  0.03 | 0.07  -0.06  0.07  0.04  0.01 |
| Impact on commuting method | 0.03 | **0.54** | -0.11 |
| Interferes with commuting | 0.24 | **0.50** | -0.12 |
| Work seriously affected by pain | 0.32 | **0.47** | 0.04 |
| Work slightly affected by pain | 0.22 | **0.48** | 0.00 |
| Psychological/cognitive impacts:  Concentration  Irritability  Make mistakes  Worry about making mistakes  Work takes longer | 0.09  0.15  -0.05  0.13  0.20 | **0.74**  **0.45**  **0.82**  **0.72**  **0.56** | 0.06  0.12  -0.07  -0.04  0.02 |
| Concern about letting colleagues down | 0.29 | **0.48** | 0.03 |
| Adequate schedule-based job modifications | 0.21 | -0.15 | **0.70** |
| Support from employer/line manager for modifications | 0.23 | -0.15 | **0.66** |
| Work has meaning | -0.38 | 0.35 | **0.49** |
| Work helps distract from pain | -0.45 | 0.29 | **0.48** |

# Supplementary File Table 5: Extracted factor correlations

|  | **Factor 1** | **Factor 2** |
| --- | --- | --- |
| **Factor 1** | 1 |  |
| **Factor 2** | 0.77 | 1 |
| **Factor 3** | 0.33 | 0.34 |

# Supplementary File Figure 1: Eigenvalues of factors used to inform factor retention


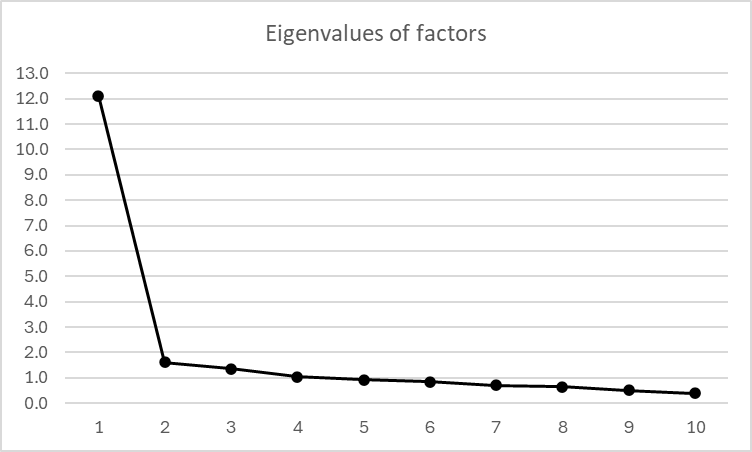


Eigenvalue

Factor
