# Supplementary material for: Development and Validation of the ‘Working With Chronic Pain: Assessment of Impacts’ (WORC‐PAIN) Questionnaire for People in Paid Work With Chronic Pain
Source: Eur J Pain. 2026 Jul 29;30(7):e70337. doi: 10.1002/ejp.70337 (PMC13420923; doi:10.1002/ejp.70337)
Supplement: Supplementary file 2 — Data S1: WORC‐PAIN_GRAMMS checklist. [file EJP-30-0-s001.docx]

Submission EURJPAIN-D-25-00983

Development and validation of the “Working with Chronic Pain: Assessment of Impacts” (WORC-PAIN) questionnaire for people in paid work with chronic pain European Journal of Pain

Reporting Checklist: Good Reporting of a Mixed-Methods Study (GRAMMS)

| GRAMMS Checklist | **Manuscript location** |
| --- | --- |
| 1. Describe the justification for using a mixed methods approach to the research question. | Introduction pg. 2 |
| 2. Describe the design in terms of the purpose, priority and sequence of the methods. | Methods pg. 2; Figure 1 |
| 3. Describe each method in terms of sampling, data collection and analysis. | Methods Phases 1-3 |
| 4. Describe where integration has occurred, how it has occurred and who has participated in it. | Figure 1; Methods pg. 5, 10; Results pg. 11 |
| 5. Describe any limitation of one method associated with the present of the other method. | Discussion pg. 17 |
| 6. Describe any insights gained from mixing or integrating methods. | Discussion pg. 16, 17, 18 |
